# Application of improved harmonic Poisson segmented regression model in evaluating the effectiveness of Kala-Azar intervention in Yangquan City, China

**DOI:** 10.3389/fpubh.2024.1326225

**Published:** 2024-07-31

**Authors:** Chongqi Hao, Zhiyang Zhao, Peijun Zhang, Bin Wu, Hao Ren, Xuchun Wang, Yuchao Qiao, Yu Cui, Lixia Qiu

**Affiliations:** ^1^School of Public Health, Shanxi Medical University, Taiyuan, Shanxi, China; ^2^School of Public Health, Sun Yat-sen University, Guangzhou, Guangdong, China; ^3^Yangquan Centre for Disease Control and Prevention, Yangquan, Shanxi, China

**Keywords:** Kala-Azar, interrupted time series analysis, improved harmonic Poisson segmented regression model, effectiveness evaluation, Yangquan

## Abstract

**Background:**

The Centre for Disease Control and Prevention in Yangquan, China, has taken a series of preventive and control measures in response to the increasing trend of Kala-Azar. In response, we propose a new model to more scientifically evaluate the effectiveness of these interventions.

**Methods:**

We obtained the incidence data of Kala-Azar from 2017 to 2021 from the Centre for Disease Control and Prevention (CDC) in Yangquan. We constructed Poisson segmented regression model, harmonic Poisson segmental regression model, and improved harmonic Poisson segmented regression model, and used the three models to explain the intervention effect, respectively. Finally, we selected the optimal model by comparing the fitting effects of the three models.

**Results:**

The primary analysis showed an underlying upward trend of Kala-Azar before intervention [incidence rate ratio (IRR): 1.045, 95% confidence interval (CI): 1.027–1.063, *p* < 0.001]. In terms of long-term effects, the rise of Kala-Azar slowed down significantly after the intervention (IRR:0.960, 95%CI:0.927–0.995, *p* = 0.026), and the risk of Kala-Azar increased by 0.3% for each additional month after intervention (*β_1_* + *β_3_* = 0.003, IRR = 1.003). The results of the model fitting effect showed that the improved harmonic Poisson segmental regression model had the best fitting effect, and the values of MSE, MAE, and RMSE were the lowest, which were 0.017, 0.101, and 0.130, respectively.

**Conclusion:**

In the long term, the intervention measures taken by the Yangquan CDC can well curb the upward trend of Kala-Azar. The improved harmonic Poisson segmented regression model has higher fitting performance, which can provide a certain scientific reference for the evaluation of the intervention effect of seasonal infectious diseases.

## 1 Background

Kala-Azar, also known as visceral leishmaniasis, is a chronic endemic infectious disease caused by the invasion of Leishmania species into the human body through the bite of a sandfly and its parasitism in human macrophages (1). China declared the basic eradication of Kala-Azar in 1958 (2), however, in recent years, the number of reported cases of Kala-Azar in China’s Shanxi Province has increased year by year, ranking first in China in the number of reported cases in 2019–2020, with the city of Yangquan having the highest number of sick people (3). In response, the Yangquan Centre for Disease Prevention and Control (CDC) carried out preventive and control measures in May 2020, mainly by means of screening patients, eradicating sandflies and culling sick dogs. A simple comparison of the difference in morbidity rates before and after this intervention is not yet sufficient to make a sound evaluation of the effectiveness of this measure, so further exploration of suitable statistical methods is essential.

In recent years, interrupted time series analysis (ITSA) has often been used to quantitatively evaluate the effectiveness of public health interventions (4), which is a quasi-experimental design to evaluate the effectiveness of an intervention at a well-defined point in time (5). In interrupted time series design, segmented regression analysis is a powerful statistical method for evaluating the effects of interventions, which can reflect both short-term and long-term effects of interventions, and is able to construct different models based on different data types, such as linear segmented regression models or Poisson segmented regression models (6). In reality, the onset of some diseases is often accompanied by significant seasonality, and this seasonality can make the time series unstable and generate autocorrelation (7), and this autocorrelation may make the standard errors of the parameter estimates of the segmented regression model small, thus overestimating the effect of the intervention, and at the same time, if the morbidity rates before and after the intervention are not uniformly distributed across the months, it may also make the study results significantly biased (4). In this regard, harmonic regression models constructed using the frequency domain approach in time series analysis have been shown to be able to cope with such problems (8). In 2017, Margaret et al. used a Poisson harmonic regression model to fit and predict the seasonal cyclical component of the Ross River disease, brucellosis, and dengue sequences in Australia, and showed that this model could fit the characteristics of the data well, with better predictive performance (9). Hongjie Yu et al. used a linear regression model with harmonic terms to estimate the seasonal characteristics of influenza in 30 provinces in China from 2005 to 2011, and the results showed that the model could fit the seasonal and cyclical fluctuations present in the influenza data well (10).

Harmonic regression model can only fit the data with regular periodic fluctuations well. The model becomes inadequate when there are irregular cyclical situations of disease change, such as peaks of incidence becoming steeper and troughs of incidence being prolonged (11). We find that an improved harmonic regression model proposed by Ramanathan et al. can better fit seasonally steep peaks by introducing sine and cosine transformation functions as quadratic terms into the harmonic regression model (12). With this in mind, we constructed an improved Poisson harmonic regression model in the hope of fitting the time-series data of Kala-Azar more accurately, and thus scientifically evaluating the effectiveness of the interventions.

In this study, we constructed an improved Poisson harmonic regression model for the first time and used it for data fitting and evaluation of interventions for Kala-Azar. We first constructed a Poisson segmented regression model as a basic model, then constructed a Poisson harmonic segmented regression model and an improved Poisson harmonic segmented regression model to cope with the seasonal cyclical condition of the time series. Finally, through the comparison of the three models, the optimal model was selected to accurately evaluate the intervention effect of Kala-Azar, which can provide a certain scientific reference for the evaluation of the intervention effect of seasonal infectious diseases.

## 2 Materials and methods

### 2.1 Data sources

The data were obtained from the Yangquan CDC, spanning from 1 January 2017 to 31 December 2021, and contained the number of confirmed local cases of Kala-Azar reported in Yangquan and related population data. The diagnosis of Kala-Azar was made with reference to the Diagnostic Criteria for Kala-Azar (WS 258–2006), and all cases were identified according to the time of onset of the disease (13), and the time-series indicator used was the incidence rate. The interval unit of the time series is month, a total of 60 months, and the intervention point is May 2020.

### 2.2 Analysis of Kala-Azar sequence characteristics and stationarity test

Seasonal-trend decomposition using Loess (STL) can be used to analyze the long-term trend, seasonal trend and random effect of Kala-Azar incidence in Yangquan from 2017 to 2021 as follows (Equation 1) (14):


(1) Xt=Tt+St+It

where *X*_t_ is the actual value of Kala-Azar incidence at time t and *T*_t_, *S*_t_ and *I*_t_ are the long-term trends, seasonal trends and random effects, respectively. The augmented Dickey–Fuller test (ADF) test was used to evaluate the stationarity of the sequence.

### 2.3 Poisson segmented regression model

The general expression of the model is as follows:


(2) $$\log\left(y_{i}\right)=\log\left(n_{i}\right)+\beta_{0}+\beta_{1}X_{1}+\beta_{2}X_{2}+\beta_{3}X_{3}+\varepsilon$$

Since the number of observed populations varies from year to year, *log(n_i_)* is introduced here as an offset to remove the effect of unequal number of observation units on the results, where *n_i_* is the number of observed populations per year. The dependent variable *y_i_* is the number of Kala-Azar cases per month, and *X_1_*, *X_2_,* and *X_3_* denote time variables, intervention variables, and post-intervention time variables, respectively. *β_0_* is the pre-intervention intercept, indicating the pre-intervention baseline level; *β_1_* is the pre-intervention trend parameter, that is, the slope of the pre-intervention regression line; *β_2_* is the immediate change parameter, that is, the difference between the estimated values of the indicators in the two regression models before and after the intervention at the moment of the intervention, which indicates the change in the level of the indicator values caused by the intervention and is used for evaluating the short-term effect of the intervention; *β_3_* is the amount of change in the trend parameter, which describes the difference between the pre and post-intervention slopes, and is used for evaluating the long-term effect of the intervention; and indicates the random error. Since June–September of each year is the peak period of sandflies, Yangquan City carried out measures to eliminate sandfly during this period. In order to evaluate the effectiveness of this measure, this paper introduces the dummy variable *X_4_*, and the time point during the elimination period is set to 1, and the rest of the time point is set to 0. Therefore, the model (2) can be changed to:


(3) $$\log\left(y_{i}\right)=\log\left(n_{i}\right)+\beta_{0}+\beta_{1}X_{1}+\beta_{2}X_{2}+\beta_{3}X_{3}+\beta_{4}X_{4}+\varepsilon$$

The parameter *β_4_* indicates the amount of level change caused by elimination of sandfly.

### 2.4 Harmonic Poisson segmental regression model

The expression of the underlying model describing the cyclical fluctuations of the time series is (15):

(4) $$y_{t}=\mu +\gamma \cos\left(2\pi \omega t+\varphi \right)+\varepsilon$$

*y_t_* is the value at moment *t*, *t* = 1, 2, …, N, N is the effective length of the time series, *μ* is a constant, *γ* is the amplitude, *ω* is the frequency, *φ* is the phase angle, and *ε* denotes the random error. Assuming that the period of the sequence is known, the frequency *ω*, is a fixed number, that is, the reciprocal of the period. Thus, the model consists of three unknown parameters: the constant *μ*, the amplitude *γ*, and the phase angle *φ*. Model (4) can be transformed into:


(5) $$\begin{array}{l} y_{t}=\mu +\gamma \cos\left(2\pi \omega t+\varphi \right)+\varepsilon =\mu +\beta_{C}\cos\left(2\pi \omega t\right)+\\ \beta_{S}\sin\left(2\pi \omega t\right)+\varepsilon \end{array}$$

$\beta_{C}=\gamma \cos\varphi$ and $\beta_{S}=-\gamma \sin\varphi$ are the coefficients of the model’s sine and cosine function terms, respectively. The time series interval unit used in this study is month and the cycle length is 12, which shows that *ω* = 1/12. Therefore, model (5) can be rewritten as:


(6) $$y_{tm}=\beta_{0}+\beta_{C}\cos\left(2\pi t_{m}/12\right)+\beta_{S}\sin\left(2\pi t_{m}/12\right)+\varepsilon$$

*y_tm_* is the monthly reported incidence value for month *t* of year *m*; *t* values range from 1 to 12; *m* ranges from 1 to L, and L is the maximum number of years of observation. The model is a linear combination of the sine and cosine functions, which fit the sequence into a single, equally spaced variation of regular fluctuations. The peak time *θ* can be estimated using *β_c_* and *β_s_* by the δ method: $\theta =12\left\{\arctan\left(\beta_{S}/\beta_{C}\right)+k\right\}/2\pi$ , When *β_c_* > 0 and *β_s_* > 0, *k* = 0; when *β_c_* < 0 and *β_s_* < 0, *k* = 2*π*; in the remaining cases *k* = *π* (12). We refer to model (6) on the basis of model (3) so as to construct the harmonic-based Poisson segmented regression model:


(7) $$\begin{array}{l} \log\left(y_{tm}\right)=\log\left(n_{m}\right)+\beta_{0}+\beta_{1}X_{1}+\beta_{2}X_{2}+\beta_{3}X_{3}+\beta_{4}X_{4}+\\ \beta_{C}\cos\left(2\pi t_{m}/12\right)+\beta_{S}\sin\left(2\pi t_{m}/12\right)+\varepsilon \end{array}$$

### 2.5 Improved harmonic Poisson segmented regression model

We use two wave functions $2\left\{1-\cos\left(u\right)\right\}/u^{2}$ and $\sin\left(u\right)/u$instead of the cosine and sine functions, respectively, so as to better fit the seasonally steep peaks. The model is constructed as follows:


(8) $$y_{tm}=\beta_{0}+\beta_{\mathrm{wave}1}\left[2\left(\begin{array}{l} 1-\\ \cos u_{m} \end{array}\right)/{u_{m}}^{2}\right]+\beta_{\mathrm{wave}2}\left[\begin{array}{l} \left(\sin u_{m}\right)/\\ u_{m} \end{array}\right]+\varepsilon$$

$u_{m}=2\pi \left(t_{m}-\theta \right)/12$, *θ* is the time of the peak and can be calculated using the δ method described above, *β*_wave1_ and *β*_wave2_ are the coefficients of the two wave functions. The improved harmonic Poisson segmented regression model we constructed on this basis is as follows:


(9) $$\begin{array}{l} \log\left(y_{tm}\right)=\log\left(n_{m}\right)+\beta_{0}+\beta_{1}X_{1}+\beta_{2}X_{2}+\beta_{3}X_{3}+\\ \beta_{4}X_{4}+\beta_{\mathrm{wave}1}\left[2\left(1-\cos u_{m}\right)/{u_{m}}^{2}\right]+\\ \beta_{\mathrm{wave}2}\left[\left(\sin u_{m}\right)/u_{m}\right]+\varepsilon \end{array}$$

### 2.6 The construction process of the models

**Figure 1:**
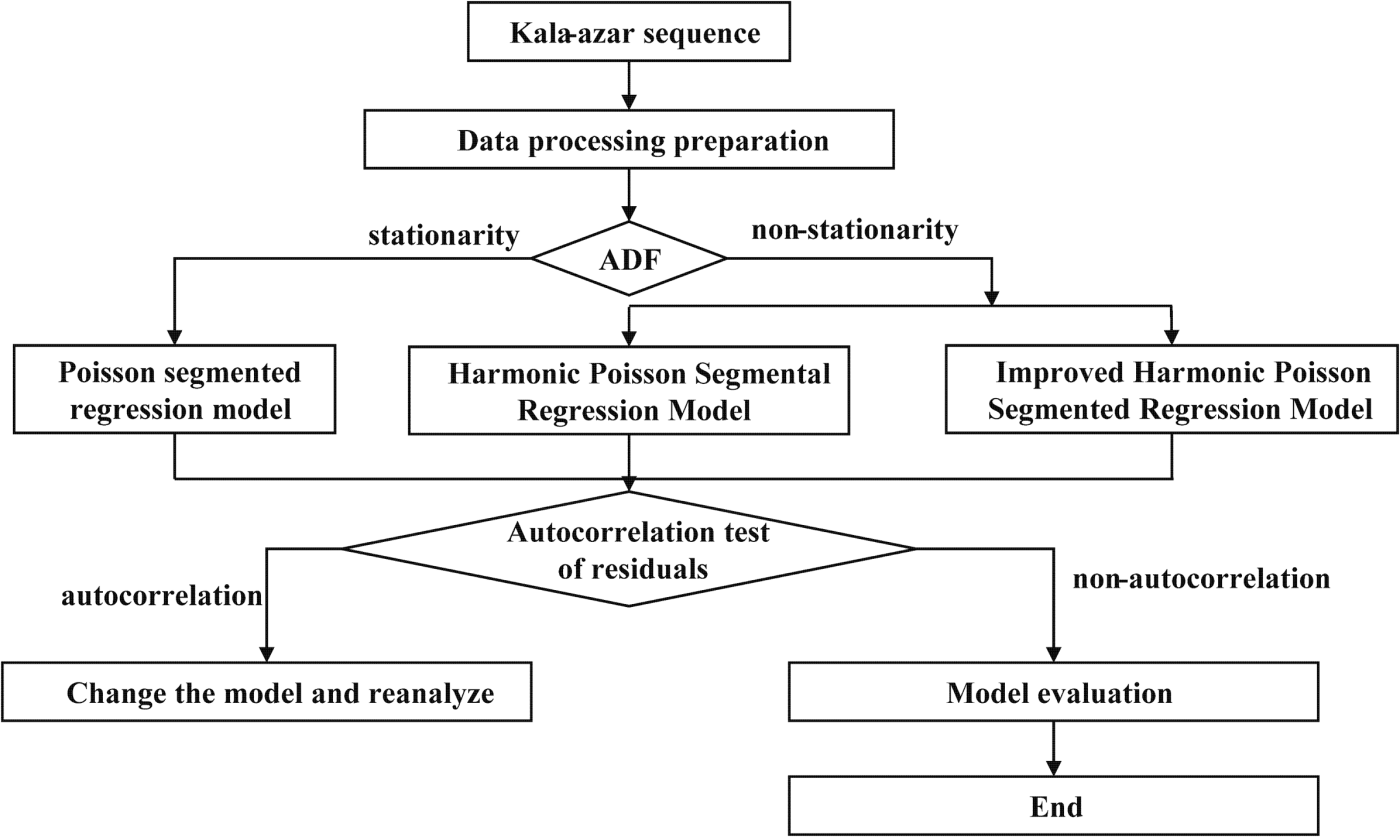
Flow chart of the three Poisson segment regression models.

Figure 1 showed the construction framework of the above three models, including five parts: data processing preparation, stationarity test, model construction, autocorrelation test of residuals and model evaluation.

#### 2.6.1 Data processing preparation

The variable assignments of the data were shown in Supplementary Table S1, in which *X_1_* represents the time variable, which in this study is sequentially from 1 to 60; *X_2_* represents the intervention variable, which is 0 before the intervention, and then 1 after; *X_3_* represents the post-intervention time variable, which is 0 before the intervention, and then 0–19 sequentially; and *X_4_* is a dummy variable, which is set to be 1 for the month of the elimination of the sandfly, and 0 for the rest of the months.

#### 2.6.2 Stationarity test

ITSA required sequence stationarity, and ADF test was used to estimate the stationarity of the sequence.

#### 2.6.3 Model construction

When the series is stationary, the Poisson segmented regression model is established by formula 3. When the sequence is non-stationarity, harmonic Poisson segmental regression model and improved harmonic Poisson segmental regression model are established by formulas 7, 9 respectively.

#### 2.6.4 Autocorrelation test of residuals

The Ljung-Box test was used to evaluate whether the residual was an autocorrelation sequence, and non-autocorrelation showed that the model was successfully constructed. In the Ljung-Box test, the null hypothesis is that “the residuals are non-autocorrelated.” The autocorrelation was double-checked by examining the autocorrelation function plots.

#### 2.6.5 Model evaluation

Mean Squared Error (MSE), Mean Absolute Error (MAE) and Root Mean Squared Error (RMSE) were used to compare the fitting performance of each model.

### 2.7 Statistical analysis

Microsoft Excel 2021 was used to collate the data and SAS 9.4 was used to implement the construction of the three models described above. Anaconda software version 4.10.3 was used for stationarity and autocorrelation test. The incidence rate ratio (IRR) and 95% confidence interval (CI) were calculated. A *p* < 0.05 for two-tailed tests indicated statistical significance.

## 3 Results

### 3.1 Sequence characteristics and stationarity test

**Figure 2:**
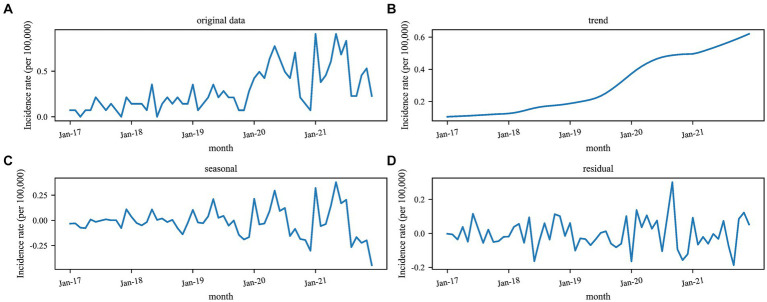
Seasonal decomposition of monthly Kala-Azar in Yangquan from 2017 to 2021. The unit is incidence per 100,000 population. **(A–D)** The original data, long-term trends, seasonal trends, and residuals, respectively.

**Figure 3:**
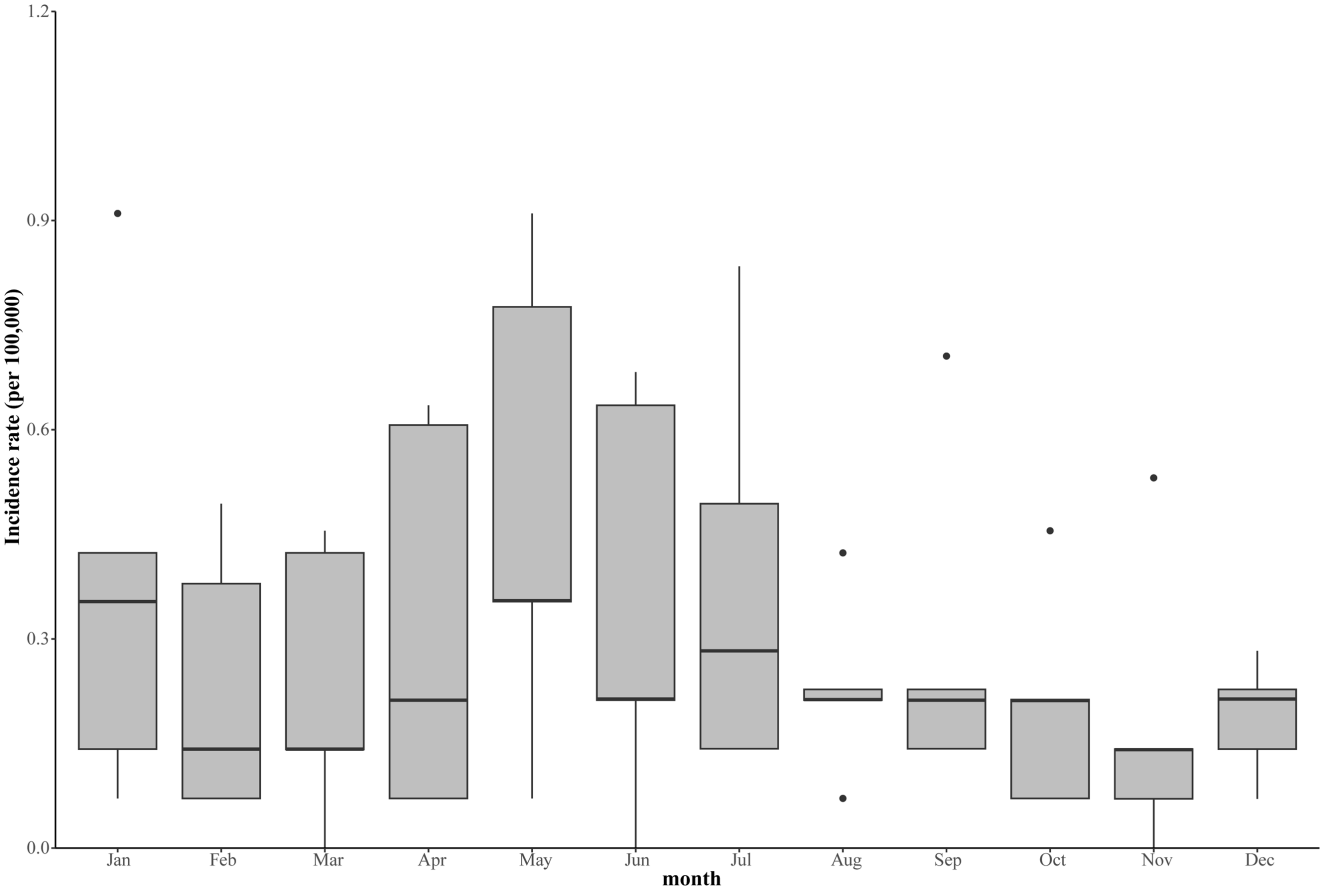
The seasonal distribution of monthly Kala-Azar in Yangquan from 2017 to 2021.

STL was used to study the time series of Kala-Azar in Yangquan from 2017 to 2021, and the results were shown in Figure 2. The long-term trend showed that the incidence of Kala-Azar in Yangquan had gradually increased since 2017 and reached its peak in mid-2020. Kala-Azar had a distinct seasonality, with the peak onset in May each year (Figures 2, 3). The stationarity test result showed that the Kala-Azar sequence was non-stationary (the ADF test: *t* = −0.403, *p* = 0.910).

### 3.2 Model construction and intervention effect evaluation

Table 1 showed the results of the three models constructed by using formulas 3, 7, and 9 respectively. The primary analysis showed an underlying upward trend of Kala-Azar before intervention (Model A: IRR:1.045, 95%CI:1.026–1.064, *p* < 0.001; Model B: IRR:1.045, 95%CI:1.027–1.064, *p* < 0.001; Model C: IRR:1.045, 95%CI: 1.027–1.063, *p* < 0.001). On top of this underlying trend, we found that in terms of long-term effects, the rise of Kala-Azar slowed down significantly after the intervention (Model A: IRR:0.947, 95%CI:0.915–0.981, *p* = 0.002; Model B: IRR:0.958, 95%CI:0.925–0.992, *p* = 0.016; Model C: IRR:0.960, 95%CI:0.927–0.995, *p* = 0.026), but the short-term effect of the intervention was not statistically significant (Model A: IRR:1.290, 95%CI:0.789–2.107, *p* = 0.310; Model B: IRR:1.208, 95%CI:0.741–1.970, *p* = 0.448; Model C: IRR:1.236, 95%CI:0.758–2.016, *p* = 0.395). The Poisson segmented regression model indicated that the risk of developing Kala-Azar decreases by 1% for each additional month after intervention (*β_1_* + *β_3_* = −0.010, IRR = 0.990). The harmonic Poisson segmental regression model showed that the risk of Kala-Azar increased by 0.1% for each additional month after intervention (*β_1_* + *β_3_* = 0.001, IRR = 1.001). Although in the opposite direction to the results obtained from the Poisson segmented regression model, the trend of Kala-Azar was significantly slower after the intervention compared to the pre-intervention period. The improved harmonic Poisson segmented Regression Model suggested that the risk of Kala-Azar increased by 0.3% for each additional month after intervention (*β_1_* + *β_3_* = 0.003, IRR = 1.003), the results were also contrary to the results of the Poisson segmented regression model, and larger than the results of the harmonic Poisson segmented regression model, but it also showed that the upward trend in the incidence of Kala-Azar slowed down significantly after the intervention. The results of the three models indicated that the interventions were not statistically significant (Model A: IRR:1.110, 95%CI:0.786–1.569, *p* = 0.554; Model B: IRR:1.090, 95%CI:0.704–1.687, *p* = 0.699; Model C: IRR:1.066, 95%CI:0.723–1.572, *p* = 0.748). The four parameters used to adjust seasonal periodicity were statistically significant, (Model B: IRR_sine_:1.279, 95%CI:1.030–1.588, *p* = 0.026; IRR_cosine_:0.771, 95%CI:0.623–0.953, *p* = 0.016. Model C: IRR_wave1_:6.309, 95%CI:2.584–15.402, *p* < 0.001; IRR_wave2_:0.259, 95%CI:0.102–0.656, *p* = 0.004).

**Table 1:** Effect analysis of intervention measures for prevention and control of Kala-Azar by CDC in Yangquan.

		Model A			Model B			Model C		
		*β*	IRR (95% CI)	*p*-value	*β*	IRR (95% CI)	*p*-value	*β*	IRR (95% CI)	*p*-value
Trend pre-intervention	*β_1_*	0.044	1.045 (1.026 to 1.064)	**<0.001**	0.044	1.045 (1.027 to 1.064)	**<0.001**	0.044	1.045 (1.027 to 1.063)	**<0.001**
Immediate change	*β_2_*	0.254	1.290 (0.789 to 2.107)	0.310	0.189	1.208 (0.741 to 1.970)	0.448	0.212	1.236 (0.758 to 2.016)	0.395
Change in trend post-intervention	*β_3_*	−0.054	0.947 (0.915 to 0.981)	**0.002**	−0.043	0.958 (0.925 to 0.992)	**0.016**	−0.041	0.960 (0.927 to 0.995)	**0.026**
Measures to eliminate sandfly	*β_4_*	0.105	1.110 (0.786 to 1.569)	0.554	0.086	1.090 (0.704 to 1.687)	0.699	0.064	1.066 (0.723 to 1.572)	0.748
sine	*β_S_*				0.246	1.279 (1.030 to 1.588)	**0.026**			
cosine	*β_c_*				−0.260	0.771 (0.623 to 0.953)	**0.016**			
wave1	*β_wave1_*							1.842	6.309 (2.584 to 15.402)	**<0.001**
wave1	*β_wave2_*							−1.352	0.259 (0.102 to 0.656)	**0.004**

IRR, incidence rate ratio; CI, confidence interval; Sine, cosine, wave1 and wave2 was used to adjust seasonality. Model A: Poisson segmented regression model. Model B: Harmonic Poisson Segmental Regression Model. Model C: Improved Harmonic Poisson Segmented Regression Model. Bolded values indicate *p*-values less than 0.05.

### 3.3 Comparison of model performance

Ljung-Box method and residual autocorrelation plots were used to test autocorrelation on the residual parts of the three models. The results of the Ljung-Box test indicated that autocorrelation still existed in the residuals of the Poisson segmented regression model (*χ*^2^ = 1.402, *P* < 0.001), whereas there is no autocorrelation found in the remaining two models, as shown in Table 2. The residual autocorrelation plots of the three models were shown in Supplementary Figure S1, which can be more intuitively seen that the residual series of the Poisson segmented regression model still had seasonal characteristics.

**Table 2:** Ljung-Box tests for the three model residuals.

Model	Ljung-Box	
	*χ^2^*	*P*
Poisson segment regression model	1.402	<0.001
Harmonic Poisson segment regression model	2.051	0.152
improved harmonic Poisson segment regression model	1.030	0.310

**Figure 4:**
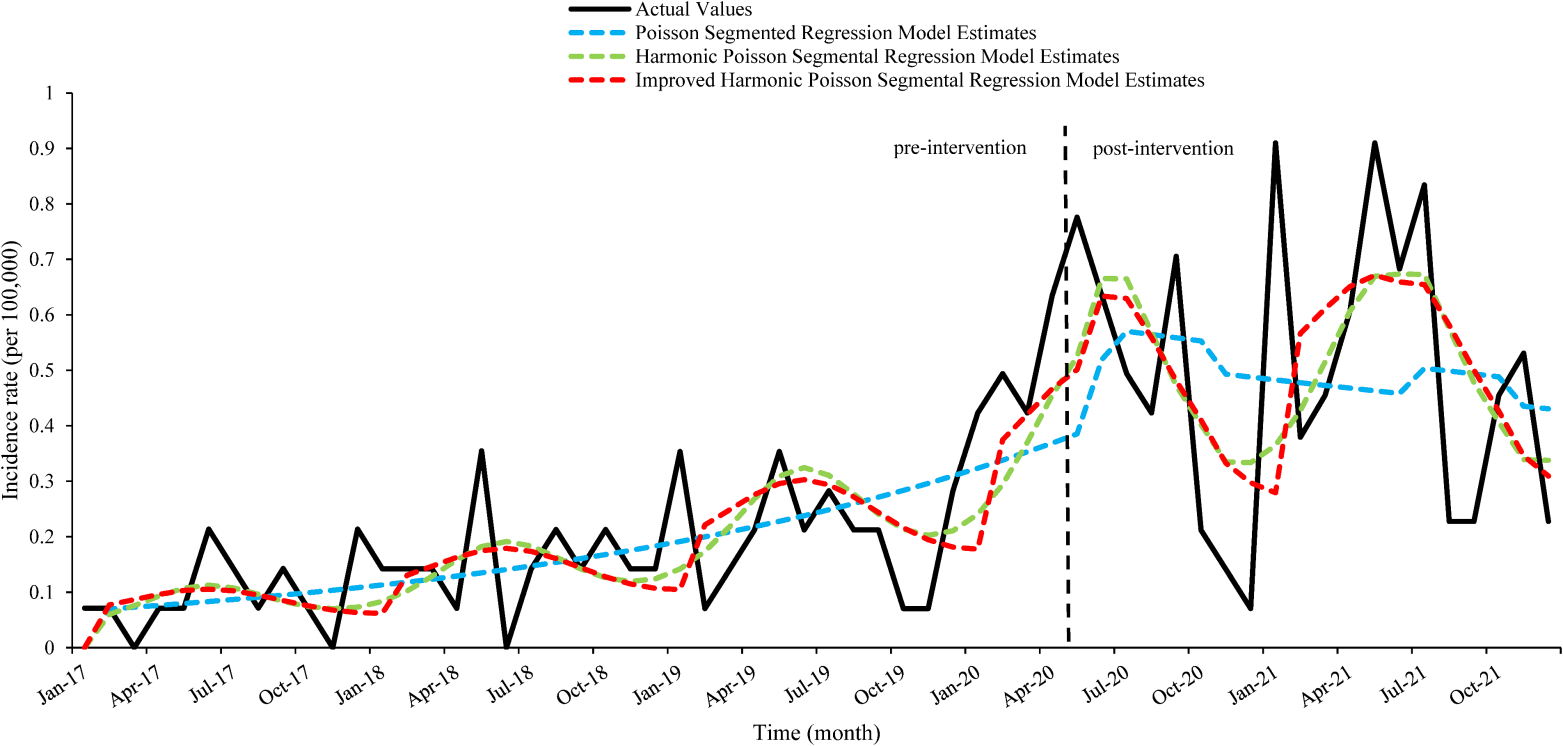
Time series plots of fitted values of the three models and actual value. The figure is divided into two parts by a dashed line. The left side of the figure is the pre-intervention part, and the right side is the post-intervention part.

In order to evaluate the fitting effect of the three models more objectively, the time series plots of the actual incidence rate and the fitted values of the three models were shown in Figure 4. MSE, MAE and RMSE were used to quantitatively compare the performance of the models (16). From Figure 4, it can be seen that the Poisson segmented regression model has the worst fitting effect; the improved harmonic Poisson segmented regression model is more advantageous in fitting the peaks and more accurately fits the trend. Among the three models, the values of MSE, MAE, and RMSE of the improved harmonic Poisson segmented regression model are the lowest, which can indicate the optimal fitting performance of the improved harmonic Poisson segmented regression model, as shown in Table 3.

**Table 3:** Performance comparison of the three models.

Model	*MSE*	*MAE*	*RMSE*
Poisson segment regression model	0.028	0.120	0.166
Harmonic Poisson segment regression model	0.019	0.103	0.138
Improved harmonic Poisson segment regression model	**0.017**	**0.101**	**0.130**

Bold indicates MSE, MAE and MRSE has the lowest value.

## 4 Discussion

As the reported incidence of Kala-Azar in Yangquan City, China, is increasing year by year and is prone to serious health service burden and economic losses, the Yangquan CDC has taken active preventive and control measures. Accurate and rational intervention effect evaluation models are important guides for infectious disease prevention, control and governmental decision-making (17). The reported incidence of Kala-Azar in Yangquan City from January 2017–December 2021 showed an increasing trend year by year, and there was a clear seasonal cyclical feature, with the peak in May each year. This may be due to the fact that May is an active period for sandfly each year, making it easier for Kala-Azar to spread, which suggests that May is an appropriate window to carry out residual spraying to control vector densities each year.

This study used a segmented regression model based on an interrupted time series design to evaluate the effect of the intervention of Kala-Azar in Yangquan City. There is obvious seasonality in the Kala-Azar sequence, which will make the time series unstable, produce autocorrelation, and make the results of the study appear obvious bias (18). In order to solve the seasonal periodicity and autocorrelation of the Kala-Azar time series, the Harmonic Poisson Segmented Regression Model, and the Improved Harmonic Poisson Segmented Regression Model were established in this paper. The results of these two models showed that the short-term effect of the intervention was poor, which may be due to the lag of intervention, it was difficult to take effect in the short term; but in the long term, the intervention curbed the onset of Kala-Azar.

Compared to the Poisson segmented regression model and the harmonic Poisson segmented regression model, the improved harmonic Poisson segmented regression model can fit the Kala-Azar sequence better. It may be due to the fact that the model uses two wave functions to simulate the seasonal cycle portion of the Kala-Azar sequence, which is better suited to seasonal steep peaks. This is also consistent with the findings of Ramanathan et al. (12).

To the best of our knowledge, we are the first one to explore the segmented regression model based on ITSA for analyzing the intervention effect of Kala-Azar in Yangquan, China. Its advantage is that the model can accurately evaluate the short-term and long-term effects of Kala-Azar intervention. Second, the harmonic Poisson segmented regression model and the improved harmonic Poisson segmented regression model established in this paper can more accurately fit the seasonal and periodic parts of the Kala-Azar sequence. At the same time, the two models can solve the autocorrelation in the sequence.

However, there are also some limitations. (1) The three models established in this study only evaluated the short-term and long-term effects of the intervention measures, but the intervention may have a certain lag, and the model could not determine the specific time when the intervention would have an effect. (2) This study only established segmented regression models, and the superiority of the improved harmonic Poisson segmented regression model and other models remained to be verified. (3) There are many factors affecting the occurrence of infectious diseases, and this study only used historical incidence data, and other factors can be included in the model for further research. In the future, the influence factors of Kala-Azar will be incorporated into the model, and we will consider the delayed effects of the intervention to determine exactly when the intervention takes effect.

## 5 Conclusion

In the long term, the intervention measures taken by Yangquan CDC can curb the upward trend of Kala-Azar. The improved harmonic Poisson segmented regression model is more suitable for seasonal infectious diseases, which can provide a certain scientific reference basis for the evaluation of the intervention effect of infectious diseases.

## Data availability statement

The data analyzed in this study is subject to the following licenses/restrictions: the data that support the findings of this study are available from the corresponding author upon reasonable request. Requests to access these datasets should be directed to LQ, qlx_1126@163.com.

## Author contributions

CH: Conceptualization, Writing – original draft, Writing – review & editing. ZZ: Methodology, Software, Writing – review & editing. PZ: Data curation, Investigation, Writing – review & editing. BW: Data curation, Investigation, Writing – review & editing. HR: Validation, Writing – review & editing. XW: Software, Validation, Writing – review & editing. YQ: Validation, Writing – review & editing. YC: Validation, Writing – review & editing. LQ: Conceptualization, Funding acquisition, Supervision, Writing – review & editing.
