# Supplementary material for: Application of improved harmonic Poisson segmented regression model in evaluating the effectiveness of Kala-Azar intervention in Yangquan City, China
Source: Front Public Health. 2024 Jul 31;12:1326225. doi: 10.3389/fpubh.2024.1326225 (PMC11322757; doi:10.3389/fpubh.2024.1326225)
Supplement: Supplementary file 1 [file Data_Sheet_1.docx]

**Table S1.** Partial data and variable assignments for segmented regression models

| Time | number of cases | Incidence rate | *X_1_* | *X_2_* | *X_3_* | *X_4_* |
| --- | --- | --- | --- | --- | --- | --- |
| January 2017 | 1 | 0.0712 | 1 | 0 | 0 | 0 |
| February 2017 | 1 | 0.0712 | 2 | 0 | 0 | 0 |
| … | … | … | … | … | … | … |
| April 2020 | 9 | 0.6349 | 40 | 0 | 0 | 0 |
| **May 2020** | **11** | **0.7760** | **41** | **1** | **0** | **0** |
| June 2020 | 9 | 0.6349 | 42 | 1 | 1 | 1 |
| … | … | … | … | … | … | … |
| May 2021 | 12 | 0.9101 | 53 | 1 | 12 | 0 |
| June 2021 | 9 | 0.6826 | 54 | 1 | 13 | 1 |
| July 2021 | 11 | 0.8343 | 55 | 1 | 14 | 1 |
| August 2021 | 3 | 0.2275 | 56 | 1 | 15 | 1 |
| September 2021 | 3 | 0.2275 | 57 | 1 | 16 | 1 |
| October 2021 | 6 | 0.4551 | 58 | 1 | 17 | 0 |
| November 2021 | 7 | 0.5309 | 59 | 1 | 18 | 0 |
| December 2021 | 3 | 0.2275 | 60 | 1 | 19 | 0 |

Incidence rates are given in units of 1 in 100,000; Bolded lines indicate intervention points.


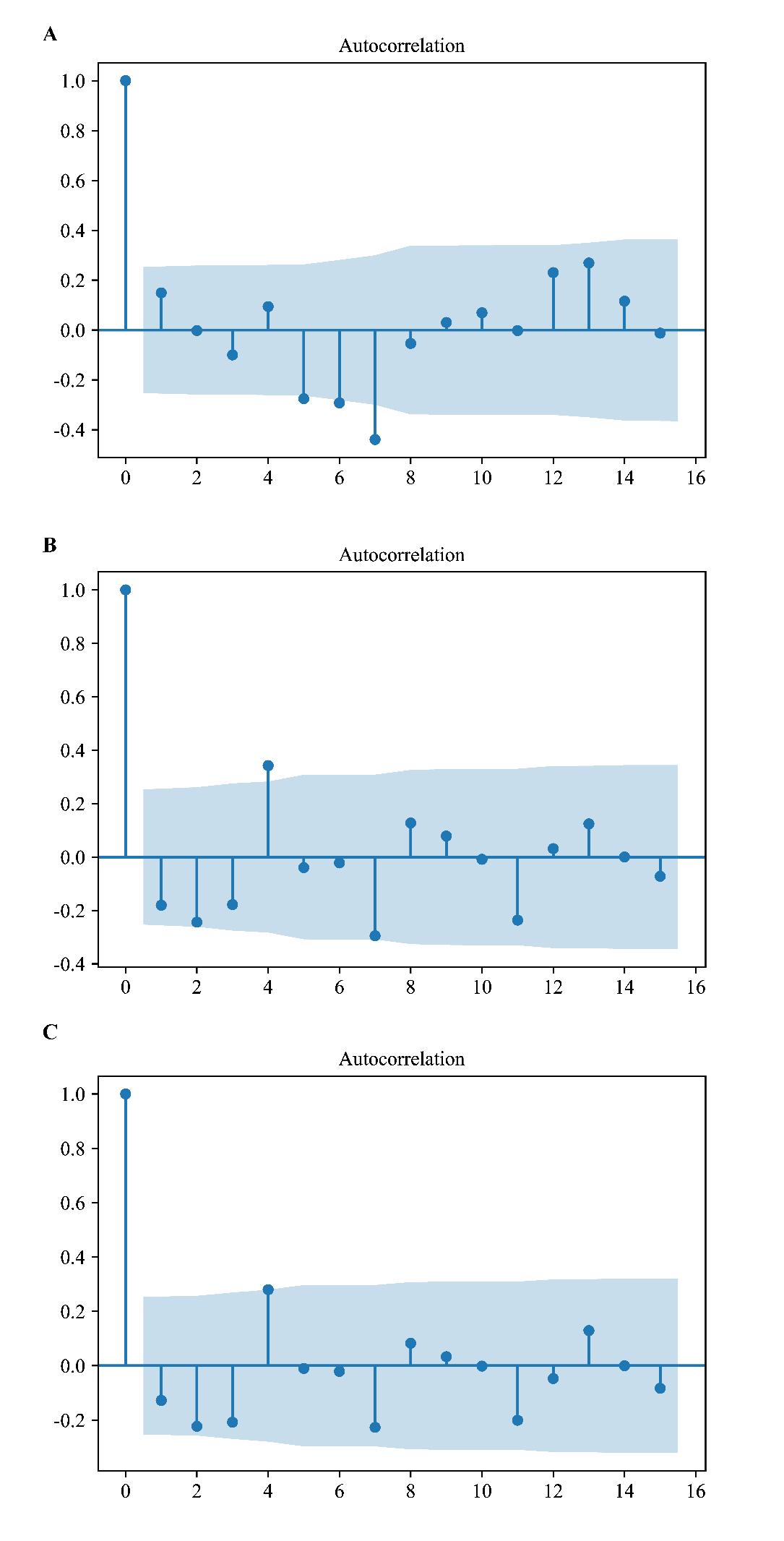


**Figure S1.** The residual autocorrelation plots of the three models.

A, B and C are the Poisson segmented regression model, Harmonic Poisson Segmental Regression Model, and Improved Harmonic Poisson Segmented Regression Model, respectively.
