# Supplementary material for: Application of improved harmonic Poisson segmented regression model in evaluating the effectiveness of Kala-Azar intervention in Yangquan City, China
Source: Front Public Health. 2024 Jul 31;12:1326225. doi: 10.3389/fpubh.2024.1326225 (PMC11322757; doi:10.3389/fpubh.2024.1326225)
Supplement: Supplementary file 2 [file Data_Sheet_2.docx]

**Poisson segmented regression model**

**proc** **import** out= WORK.its

DATAFILE= "D:\\.xlsx"

DBMS=EXCEL REPLACE;

RANGE=" $";

GETNAMES=YES;

MIXED=NO;

SCANTEXT=YES;

USEDATE=YES;

SCANTIME=YES;

**run**;

**proc** **contents** data=its;**run**;

/*log transform the standardised population*/

**data** its1;

set its;

ln=log(all);

**run**;

**proc** **genmod** data=its1;

model N= time interpret jiaohu interpret2/

link=log dist=nb offset=ln noscale;

output out=predc pred=pred;

**run**;

**proc** **genmod** data=its1;

model N= time interpret jiaohu interpret2//**/

link=log dist=poisson offset=ln ;

output out=predc pred=pred;

**run**;

**proc** **genmod** data=its1;

model N= time interpret jiaohu interpret2/

link=log dist=poisson offset=ln;

ods output Genmod.ParameterEstimates=rr;

**run**;

**data** rr1(keep=Parameter rr rr_95lower rr_95upper ProbChiSq);

retain Parameter rr rr_95lower rr_95upper ProbChiSq;

set rr;

rr=exp(Estimate);

rr_95lower=exp(LowerWaldCL);

rr_95upper=exp(UpperWaldCL);

**run**;

**proc** **print** data=rr1;**run**;

**data** its2;

set predc;

rate1=N/all***100000**;

rate2=pred/all***100000**;

**run**;

**proc** **means** data=its2;

var rate1 rate2 time;**run**;

**Harmonic Poisson Segmental Regression Model**

**data** its3;

set its2;

tsin=sin(**2***arcos(-**1**)*month/**12**);

tcos=cos(**2***arcos(-**1**)*month/**12**);

**run**;

**proc** **genmod** data=its3;

model N= time interpret jiaohu interpret2 tsin tcos/

link=log dist=nb offset=ln noscale;

output out=predc pred=pred_harmonic;

**run**;

**proc** **genmod** data=its3;

model N= time interpret jiaohu interpret2 tsin tcos/

link=log dist=poisson offset=ln;

output out=predc pred=pred_harmonic;

**run**;

**proc** **genmod** data=its3;

model N= time interpret jiaohu interpret2 tsin tcos/

link=log dist=poisson offset=ln;

ods output Genmod.ParameterEstimates=estimate_harmc;

**run**;

**data** rr_harmc(keep=Parameter rr rr_95lower rr_95upper ProbChiSq);

retain Parameter rr rr_95lower rr_95upper ProbChiSq;

set estimate_harmc;

rr=exp(Estimate);

rr_95lower=exp(LowerWaldCL);

rr_95upper=exp(UpperWaldCL);

**run**;

**proc** **print** data=rr_harmc;**run**;

**data** its6;

set predc;

rate1=N/all***100000**;

rate3=pred_harmonic/all***100000**;

**run**;

**Improved Harmonic Poisson Segmented Regression Model**

**data** estimate_sin(keep=Estmat_sin);

set estimate_harmc;

Estmat_sin=Estimate;

if Parameter='tsin' then output;

**run**;

**data** estimate_cos(keep=Estmat_cos);

set estimate_harmc;

Estmat_cos=Estimate;

if Parameter='tcos' then output;

**run**;

**data** estimate;

merge estimate_sin estimate_cos;

cita=**12***(atan(Estmat_sin/Estmat_cos)+**0**)/(**2***arcos(-**1**));

**run**;

**data** its4;

merge predc estimate;

retain cita1;

cita1=sum(cita,cita1);

u=**2***arcos(-**1**)*(month-cita1)/**12**;

foutr_cos=(sin(u/**2**)/(u/**2**))****2**;

foutr_sin=sin(u)/u;

rate3=pred_harmonic/all***100000**;

drop cita Estmat_sin Estmat_cos;

**run**;

**proc** **genmod** data=its4;

model N= time interpret jiaohu interpret2 foutr_cos foutr_sin/

link=log dist=nb offset=ln noscale;

output out=predc pred=pred_fourtier;

**run**;

**proc** **genmod** data=its4;

model N= time interpret jiaohu interpret2 foutr_cos foutr_sin/

link=log dist=nb offset=ln;

output out=predc pred=pred_fourtier;

**run**;

**proc** **genmod** data=its4;

model N= time interpret jiaohu interpret2 foutr_cos foutr_sin/

link=log dist=nb offset=ln;

ods output Genmod.ParameterEstimates=estimate_fouter;

**run**;

/*output the RR and 95%CI after adjusting for seasonality*/

**data** rr_fouter(keep=Parameter rr rr_95lower rr_95upper ProbChiSq);

retain Parameter rr rr_95lower rr_95upper ProbChiSq;

set estimate_fouter;

rr=exp(Estimate);

rr_95lower=exp(LowerWaldCL);

rr_95upper=exp(UpperWaldCL);

**run**;

**proc** **print** data=rr_fouter;**run**;

**data** its5;

set predc;

rate4=pred_fourtier/all***100000**;

**run**;

**Stationarity and autocorrelation test**

import pandas as pd

from matplotlib.pyplot import MultipleLocator

from statsmodels.graphics.tsaplots import plot_acf

from statsmodels.graphics.tsaplots import plot_pacf

#Read data

excelFile ='D:/.xlsx'

data = pd.read_excel(excelFile,sheet_name=" ", usecols=['time', 'non'],index_col = 'time')

a = pd.DataFrame(data,dtype=np.float64)

#Draw autocorrelations

font1 = {'family': 'SimSun','size':11}

font2 = {'family': 'Times New Roman','size':11}

plot_acf(data,lags=15).show()

plt.xticks(FontProperties=font2)

plt.yticks(FontProperties=font2)

plt.xlabel('lag',font1)

plt.ylabel('ACF',font2)

plt.title(' autocorrelations ',font1)

plt.savefig(r"D:/.jpg", dpi=300)

plt.show()

#Draw partial autocorrelations

plot_pacf(data,lags=15).show()

plt.xticks(FontProperties=font2)

plt.yticks(FontProperties=font2)

plt.xlabel(' lag ',font1)

plt.ylabel('PACF',font2)

plt.title(' partial autocorrelations ',font1)

plt.savefig(r"D:/.jpg", dpi=300)

plt.show()

# ljungbox test

x1 = np.array(data['non'])

from statsmodels.stats.diagnostic import acorr_ljungbox

series = x1

print(u' ljungbox_results：',acorr_ljungbox(x1, lags= 1))
